# The Effects of Viewing Cute Pictures on Performance During a Basketball Free-Throw Task

**DOI:** 10.3389/fpsyg.2021.610817

**Published:** 2021-04-26

**Authors:** Naoki Yoshikawa, Hiroaki Masaki

**Affiliations:** Faculty of Sport Sciences, Waseda University, Saitama, Japan

**Keywords:** choking under pressure, clutch, cuteness-viewing effect, Kawaii, basketball free-throw

## Abstract

Previous studies have shown that viewing cute pictures leads to performance improvement in a subsequent fine motor task. We examined the beneficial effects of viewing cute pictures in a more complex sporting skill (i.e., basketball free throws) by comparing three conditions (viewing baby animal pictures, adult animal pictures, and no pictures) and two tests (no-pressure and pressure). The participants, all of whom were college basketball players, performed 16 free throws in each condition. In the no-pressure test, male participants improved performance after viewing pictures of baby animals but not after adult animals and no pictures. In the pressure test, no significant improvement was observed. For female participants, the cuteness-viewing effect was not observed in both tests. The results suggest that viewing cute pictures may improve performance during basketball free throws in a low-pressure situation by narrowing the breadth of attentional focus and inducing approach motivation and caregiving behaviors.

## Introduction

It is well known that when attempting a basketball free-throw, the player must induce focal attention toward the hoop (i.e., external attention) to achieve success under pressure (e.g., Vickers, 1996; Wulf, 2013). Discovery of an effective and efficient method to facilitate attention control and narrow attentional focus toward the hoop may help basketball players to increase their free-throw success rate. This study tested one potential method (i.e., viewing cute pictures) in a basketball free-throw task.

Recent studies have confirmed that viewing cute pictures of puppies and kittens induces focal attention and results in performance improvement in subsequent cognitive or motor tasks (Sherman et al., 2009; Nittono et al., 2012; Yoshikawa et al., 2020). However, there is no such effect after viewing pictures of adult dogs and cats. These studies involved a fine motor task in which the participant used tweezers to pick small objects out of individual holes on a gameboard. The results consistently showed that manual dexterity—which was reflected in the number of successful pick-ups—improved after viewing pictures of baby animals. However, the “cuteness-viewing” effect is not limited to enhancing performance in fine motor tasks. Nittono et al. (2012) found that after viewing pictures of baby animals, the number of correct trials increased in a visual search task, suggesting enhanced carefulness in the perceptual domain, while the global precedence effect decreased in a global-local letter task (i.e., an increase in paying attention to details). According to these findings, theoretically, it is reasonable to presume that viewing cute pictures also improves any sporting skill that requires attentional focus.

The beneficial effect of viewing cuteness has been explained through the cognitive and affective-motivational aspects of brain function. In terms of the cognitive aspect, the beneficial effect can be attributed to both bottom-up and top-down attention control (Yoshikawa et al., 2020). In fact, cuteness embedded in visual stimuli arouses attention via bottom-up processes at a very early stage (Brosch et al., 2007, 2008; Lucion et al., 2017). In addition, viewing cuteness appears to sustain the enhanced attentional state for several seconds (Nittono and Ihara, 2017), during which executing a motor task may result in better results via top-down processing (Sherman et al., 2009; Nittono et al., 2012; Yoshikawa et al., 2020). In both cases, viewing cuteness facilitates attention control and narrows the breadth of attentional focus (Gable and Harmon-Jones, 2008; Nittono et al., 2012; Noguchi and Tomoike, 2016). In terms of affective-motivational aspects, it has been reported that viewing cuteness induces approach motivation (Glocker et al., 2009), increases physical tenderness during execution of a motor task (Sherman et al., 2009), and promotes caregiving behavior (Griskevicius et al., 2010), all of which increase performance accuracy.

Although previous studies have corroborated the beneficial aftereffects of viewing cuteness in laboratory experiments using fine motor tasks and cognitive tasks, it is still unknown whether such findings are applicable to more complex and real sporting tasks. Nevertheless, viewing cuteness would be a more pragmatic and efficient way to enhance performance in sporting events compared to special trainings and interventions. Therefore, we examined the effects of viewing baby animals on basketball free-throw performance, wherein players are required to adequately gaze at and attend to the hoop before throwing the ball to achieve success (e.g., Vickers, 1996). Among various sporting skills, the basketball free-throw can be tested under controlled situations by fixing the shooting distance and direction. It also requires attentional focus that underpins the cuteness-viewing effect. In this study, we tested experienced basketball players to exclude factors associated with premature motor skills. We recruited basketball players from limited populations of a university. Because we found differences in competitive skill level and trait anxiety between male and female teams, we included team as a group factor for statistical analyses.

Given that viewing cute pictures induces approach motivation and attentional focus, it would also be an effective and efficient tool to prevent performance deterioration during sporting games (i.e., choking under pressure; Baumeister, 1984). A plausible explanation for the underlying mechanisms of choking is that the inward attention caused by an anxiety-inducing situation (e.g., the existence of an audience) impairs the automaticity of fully learned movements and gives rise to performance deterioration even among expert athletes (see also Wine, 1971; Baumeister, 1984; Eysenck and Calvo, 1992 for different perspectives on choking mechanisms; Masters, 1992; Beilock and Carr, 2001). Hence, enhancing attention control toward the target object would restore the deteriorated performance.

Yoshikawa et al. (2020) reported that in a fine motor task, viewing pictures of baby animals increased performance accuracy and prolonged gaze duration on the hoop in a no-pressure test, while it only increased performance accuracy in a pressure test. Since manipulating pressure did not increase anxiety, it is unclear whether viewing cute pictures can prevent performance deterioration in a pressure situation. Further research is needed to confirm whether viewing cuteness can mitigate or prevent choking under pressure. Thus, we compared the effects of viewing cute pictures using no-pressure and pressure tests. We also considered the difference in competitive skill level between female and male participants to test the cuteness-viewing effect, although the statistical power would be smaller in the limited sample. We hypothesized that viewing cute pictures (i.e., those of baby animals) can improve performance in a basketball free-throw task that requires attentional focus especially in a no-pressure situation, and even prevent performance deterioration in a pressure situation.

## Materials and Methods

### Participants

Twenty-six basketball players (12 men and 14 women, *M* age = 20.6, *SD* = 1.2) who belonged to University teams participated in two sessions each (no-pressure test and pressure test). The mean years of experience was 12.8 years (*SD* = 2.4) and 12.6 years (*SD* = 1.6) for the male and female teams, respectively. The female team had won first place in the Kanto regional league matches for the last 5 years. Meanwhile, the male team placed eighth for the last 2 years. One male participant was excluded from the analysis due to his misunderstanding of the procedure in the pressure test. We applied G^*^Power 3.1 to a power analysis (Faul et al., 2007)^1^, revealing 18 as the necessary number (medium power). According to this calculation, our sample size of 25 met this criterion, showing a medium effect size. Nittono et al. (2012) and Yoshikawa et al. (2020) also reported medium, or even larger, effect sizes (η_*p*_^2^) (0.17 and 0.12, respectively) for interactions.

### Ethical Considerations

Written informed consent was obtained from all participants. After the experiment, participants were debriefed regarding the purpose of the study. The study procedure was approved by the Waseda University Ethics Committee.

### Task

We used a basketball free-throw task wherein participants shot a ball (official size of six for women and seven for men) from the standard distance (i.e., 4.2 m) to a regular hoop mounted at the standard height (i.e., 3.05 m) from the ground. One of the experimenters stood by the hoop and afforded a ball to the participant at each trial.

### Procedure

The recruited athletes participated in two sessions (i.e., no-pressure test and pressure test) that were conducted on different days (the interval between the sessions differed among participants, ranging from 1 to 13 days), except for four participants who conducted both tests on the same day at 4-h intervals due to their schedule. Three conditions (i.e., picture of a baby animal, picture of an adult animal, and no picture) were applied in each test in the same order. The order of the conditions as well as the order of the two tests were counterbalanced among participants.

Participants warmed up sufficiently before the tests. Prior to the execution of the free-throw task in each session, participants were instructed to view photographs of baby animals in the baby animal condition and of adult animals in the adult animal condition. No photograph was viewed in the control condition. In the baby and adult animal conditions, participants were given seven sheets (randomly selected for each participant) of paper (210 × 297 mm) that contained a colored image of an animal and were asked to rank the images according to their preference within 1.5 min. As per study procedure, they were made to look at the seven pictures before each test (Nittono et al., 2012; Yoshikawa et al., 2020). Furthermore, participants viewed one of the photographs for 10 s every two throws, presented in the default order of the picture rather than in their subjectively ranked order. In the control condition, participants were given a 15-s rest every two throws instead of viewing a picture. In total, participants performed 16 free throws under each condition. Participants had a short break (approximately 60 s) between conditions.

We used seven images of puppies and kittens in the baby animal condition, and seven images of dogs, cats, and a lion in the adult animal condition. The royalty-free images were downloaded from the Internet and were selected based on a pilot survey. In the survey, images of baby and adult animals differed in subjective rating scores for cuteness, infantility, and approach motivation (i.e., want to get closer), but did not differ in terms of pleasantness and excitement, which was in accordance with a previous study (Nittono et al., 2012). The same set of pictures was used for both no-pressure and pressure tests. After the second session, participants were asked to view the 14 pictures and rate them on 6-point Likert scales in terms of cuteness, infantility, pleasantness, excitation, and approach motivation, ranging from 1 (*not at all*) to 6 (*very much*).

### Pressure Manipulation

To increase anxiety in the pressure test, each participant paired up with another participant, and both were instructed to pressure one another. During the free throws, each counterpart watched their partner's performance from underneath the basketball hoop. Although each pair knew each other, they differed in either grade or gender. In the pressure test, participants were also informed that they would receive additional strength training depending on the number of their misses in the free-throw task. When participants finished three conditions, their counterparts subsequently executed the task.

### Recordings and Data Analysis

#### Behavioral Data

We scored the percentage of successful attempts of each participant by dividing the number of successful trials with the total number of free throws.

#### State and Trait Anxiety

Cognitive state anxiety was measured using the State-Trait Anxiety Inventory (STAI from Y-1 and Y-2; Spielberger and Gorsuch, 1983; the Japanese version was translated by Hidano et al., 2000a). The STAI consisted of 20 items that assessed participants' level of anxiety. Participants were asked to rate each item on a 4-point Likert scale ranging from 1 (*not at all*) to 4 (*very much so*). The total score for the 20 items was then used for analysis. In this study, the Japanese version of the STAI (STAI-JYZ; Hidano et al., 2000a), which considers Japanese cultural factors, was used. The participants completed the STAI form Y-1 before the start of the pressure and no-pressure tests, and the STAI form Y-2 after both sessions. To compare anxiety level between male and female participants, both state and trait anxiety scores were converted to *T*-scores based on the average and standard deviation of male and female college students (men: *n* = 1,088, women: *n* = 1,165) described in the STAI manual (Hidano et al., 2000b).

#### Statistical Analysis

Mean percent success was subjected to a three-factor mixed-design ANOVA with group (male/female) as between-subjects factor, and test (pressure/no-pressure) and picture-viewing condition (baby animals/adult animals/no picture) as within-subjects factors. We applied another three-way ANOVA in each test with group (male/female), test (pressure/no-pressure), and picture-viewing conditions (baby animals/adult animals) to mean change rates of percent success in the baby and adult animal conditions against the no picture condition.

The subjective rating score on each emotional item was subjected to a two-factor mixed-design ANOVA with group and viewing-picture condition as between- and within-subjects factors, respectively. Alternatively, the state anxiety score was subjected to a two-factor mixed-design ANOVA with group and test as between- and within-subjects factors, respectively.

When a significant main effect or an interaction associated with the picture-viewing condition was obtained, *post-hoc* mean comparisons were performed using the Bonferroni correction. There were no significant violations of sphericity; therefore, no corrections were required. An independent *t*-test was performed on the trait anxiety data (male/female). Effect sizes were calculated using partial eta squared (η_*p*_^2^) for omnibus comparisons and Cohen's *d* for simple comparisons. Statistical analyses were performed using SPSS version 25 for Mac (SPSS Inc., Chicago, IL, USA). We reported 95% Confidence Interval for Cohen's *d* calculated by the Jeffreys's Amazing Statistics Program (JASP, JASP Team., 2020).

## Results

### Subjective Ratings

Subjective ratings for each emotion item in both picture conditions are shown in Table 1. For cuteness, we found a significantly higher score for the baby (*M* ±*SEM* = 4.65 ± 0.17) than the adult animal condition (*M* ± *SEM* = 3.94 ± 0.17) [*F*_(1, 23)_ = 10.69, *p* = 0.003, η_*p*_^2^ = 0.32], no significant difference between the groups [*F*_(1, 23)_ = 0.05, *p* = 0.818, η_*p*_^2^ = 0.002], and an interaction [*F*_(1, 23)_ = 3.48, *p* = 0.075, η_*p*_^2^ = 0.13].

**Table 1:** Mean subjective rating scores of photo images.

	**Baby animals**		**Adult animals**	
	**Female team *(SEM)***	**Male team *(SEM)***	**Female team *(SEM)***	**Male team *(SEM)***
Cute	4.48 (0.25)	4.86 (0.21)	4.15 (0.22)	3.66 (0.25)
Infantile	4.85 (0.20)	5.56 (0.13)	1.91 (0.17)	1.83 (0.17)
Pleasant	4.21 (0.25)	4.62 (0.31)	3.87 (0.31)	3.29 (0.19)
Exciting	2.33 (0.27)	2.08 (0.30)	2.79 (0.28)	2.13 (0.28)
Wanting to get closer	4.42 (0.28)	3.75 (0.36)	3.95 (0.28)	2.81 (0.31)

*SEM, standard error of the mean*.

For infantility, we found main effects of picture [*F*_(1, 23)_ = 413.39, *p* < 0.001, η_*p*_^2^ = 0.95] and group [*F*_(1, 23)_ = 3.03, *p* = 0.095, η_*p*_^2^ = 0.12], and a significant interaction [*F*_(1, 23)_ = 5.78, *p* = 0.025, η_*p*_^2^ = 0.20]. Although the infantility score was higher for the baby than the adult animal condition for both male and female participants (male: *p* < 0.001, *d* = 3.54, 95% CI for Cohen's *d* [2.09, 4.97], female: *p* < 0.001, *d* = 4.71, 95% CI for Cohen's *d* [2.59, 6.82]), male participants rated baby animals higher (*M* ±*SEM* = 5.56 ± 0.13) than female participants (*M* ± *SEM* = 4.85 ± 0.20; *p* = 0.009, *d* = 1.15, 95% CI for Cohen's *d* [0.28, 2.00]).

For pleasantness, we found a significant main effect of picture condition [*F*_(1, 23)_ = 15.68, *p* = 0.001, η_*p*_^2^ = 0.41], no difference between the groups [*F*_(1, 23)_ = 0.07, *p* = 0.795, η_*p*_^2^ = 0.003], and a significant interaction [*F*_(1, 23)_ = 5.43, *p* = 0.029, η_*p*_^2^ = 0.19]. Simple effects analysis showed that male participants gave higher scores for baby animals than adult animals (*p* < 0.001, *d* = 1.25, 95% CI for Cohen's *d* [0.43, 2.04]). This was not the case for female participants (*p* = 0.23, *d* = 0.33, 95% CI for Cohen's *d* [−0.21, 0.87]).

For excitement, neither the main effect of picture condition [*F*_(1, 23)_ = 1.57, *p* = 0.223, η_*p*_^2^ = 0.06], nor of the group [*F*_(1, 23)_ = 1.69, *p* = 0.207, η_*p*_^2^ = 0.07] was found, and the interaction [*F*_(1, 23)_ = 1.00, *p* = 0.328, η_*p*_^2^ = 0.04] was not significant.

For approach motivation, the main effects of the picture condition [*F*_(1, 23)_ = 8.79, *p* = 0.007, η_*p*_^2^ = 0.28] and group [*F*_(1, 23)_ = 6.00, *p* = 0.022, η_*p*_^2^ = 0.21] were significant. The approach motivational score was higher for baby animals (*M* ±*SEM* = 4.13 ± 0.23) than adult animals (*M* ± *SEM* = 3.45 ± 0.24), and higher for female participants (*M* ± *SEM* = 4.18 ± 0.29) than male participants (*M* ± *SEM* = 3.28 ± 0.36). There was no interaction [*F*_(1, 23)_ = 1.00, *p* = 0.327, η_*p*_^2^ = 0.04].

### Percent Success

**Figure 1:**
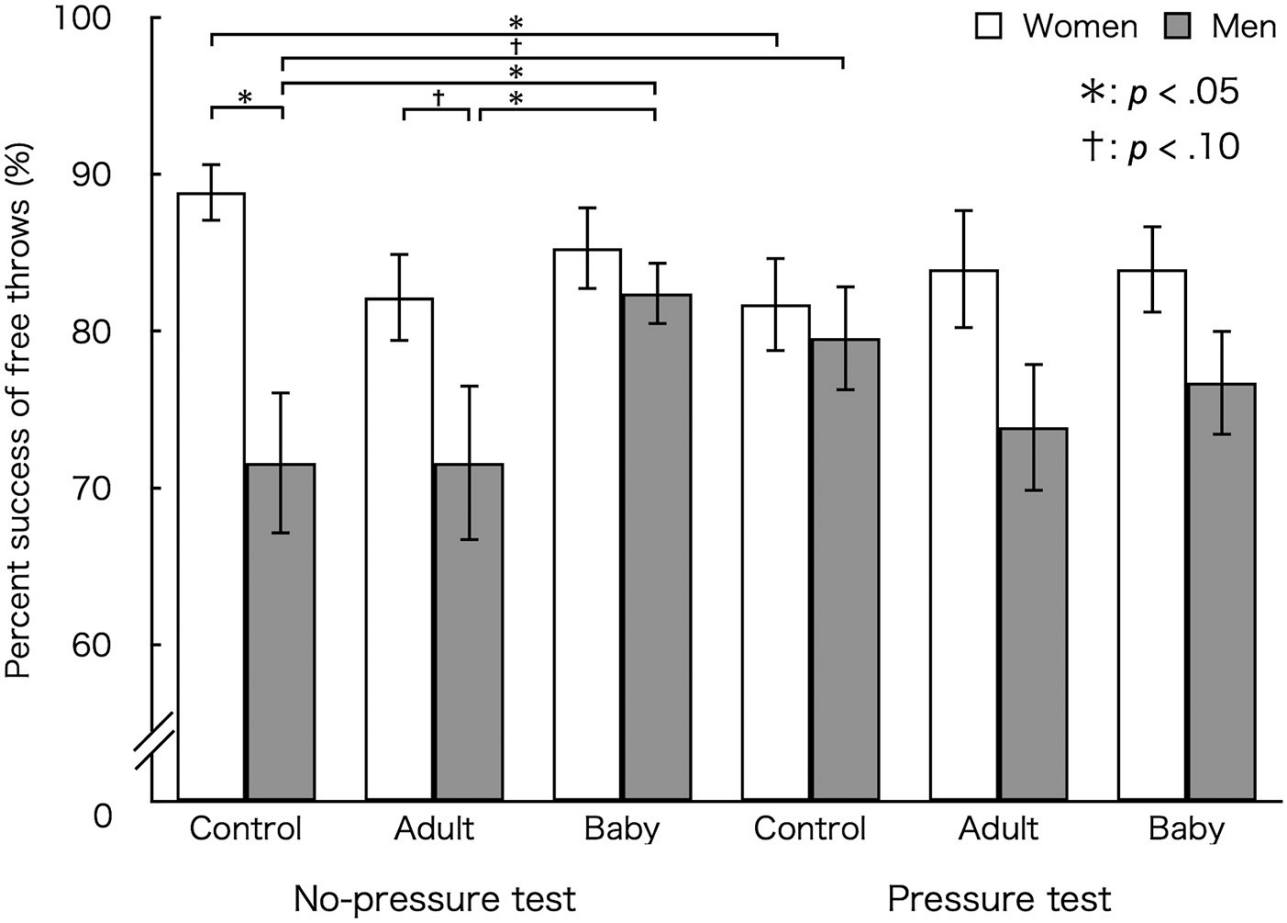
Mean percent success in each test. Baby, viewing pictures of baby animals; Adult, viewing pictures of adult animals; Control, viewing no pictures. Error bars indicate standard error of the mean (SEM).

Figure 1 shows the mean percent success. Three-way ANOVA revealed that percent success was higher for female (*M* ± *SEM* = 84.30 ± 2.12%) than male (*M* ± *SEM* = 75.95 ± 2.34%) [*F*_(1, 23)_ = 6.85, *p* = 0.015, η_*p*_^2^ = 0.23) participants. We found a main effect of picture [*F*_(2,46)_ = 2.77, *p* = 0.073, η_*p*_^2^ = 0.11] and a three-way interaction [group × test × picture: *F*_(2,46)_ = 3.73, *p* = 0.032, η_*p*_^2^ = 0.14]. Thus, we conducted the following simple effects analyses for lower level interactions and main effects.

In the control condition, a simple interaction effect of group × test [*F*_(1, 23)_ = 8.62, *p* = 0.007] was significant. In addition, percent success was lower in the pressure (*M* ±*SEM* = 81.70 ± 3.04%) than in the no-pressure test for female individuals (*M* ±*SEM* = 88.84 ± 1.87%) [*F*_(1, 23)_ = 4.39, *p* = 0.047, *d* = 0.54, 95% CI for Cohen's *d* [-0.03, 1.10]] whereas it tended to be higher in the pressure (*M* ±*SEM* = 79.55 ± 3.38%) than in the no-pressure test (*M* ± *SEM* = 71.59 ± 4.57%) for male individuals [*F*_(1, 23)_ = 4.27, *p* = 0.050, *d* = 0.65, 95% CI for Cohen's *d* (−1.29, 0.02)].

In the no-pressure test, a simple interaction effect of group × picture was also significant [*F*_(2,46)_ = 4.13, *p* = 0.022]. In addition, percent success was higher for female (*M* ±*SEM* = 88.84 ± 1.87%) than male individuals (*M* ± *SEM* = 71.59 ± 4.57%) in the control condition [*F*_(1, 23)_ = 14.37, *p* = 0.001, *d* = 1.53, 95% CI for Cohen's *d* [−0.47, 1.12]] while it tended to be higher for female (*M* ± *SEM* = 82.14 ± 2.85%) than male individuals (*M* ± *SEM* = 71.59 ± 5.01%) in the adult animal condition [*F*_(1, 23)_ = 3.72, *p* = 0.066, *d* = 0.78, 95% CI for Cohen's *d* [−0.05, 1.59]]. Furthermore, within this interaction, there was a significant main effect of picture for male individuals [*F*_(2,46)_ = 5.55, *p* = 0.007]. Bonferroni-adjusted *post-hoc* tests revealed that the percent success of male individuals was higher in the baby animal condition (*M* ±*SEM* = 82.39 ± 2.03%) than in the adult animal (*M* ± *SEM* = 71.59 ± 5.01%, *p* = 0.036, *d* = 0.86, 95% CI for Cohen's *d* [−0.15, 1.55]) and control conditions (*M* ± *SEM* = 71.59 ± 4.57%, *p* = 0.024, *d* = 0.86, 95% CI for Cohen's *d* [−0.15, 1.55]). There was a simple interaction effect of test × picture for male individuals, [*F*_(2,46)_ = 3.05, *p* = 0.057].

**Figure 2:**
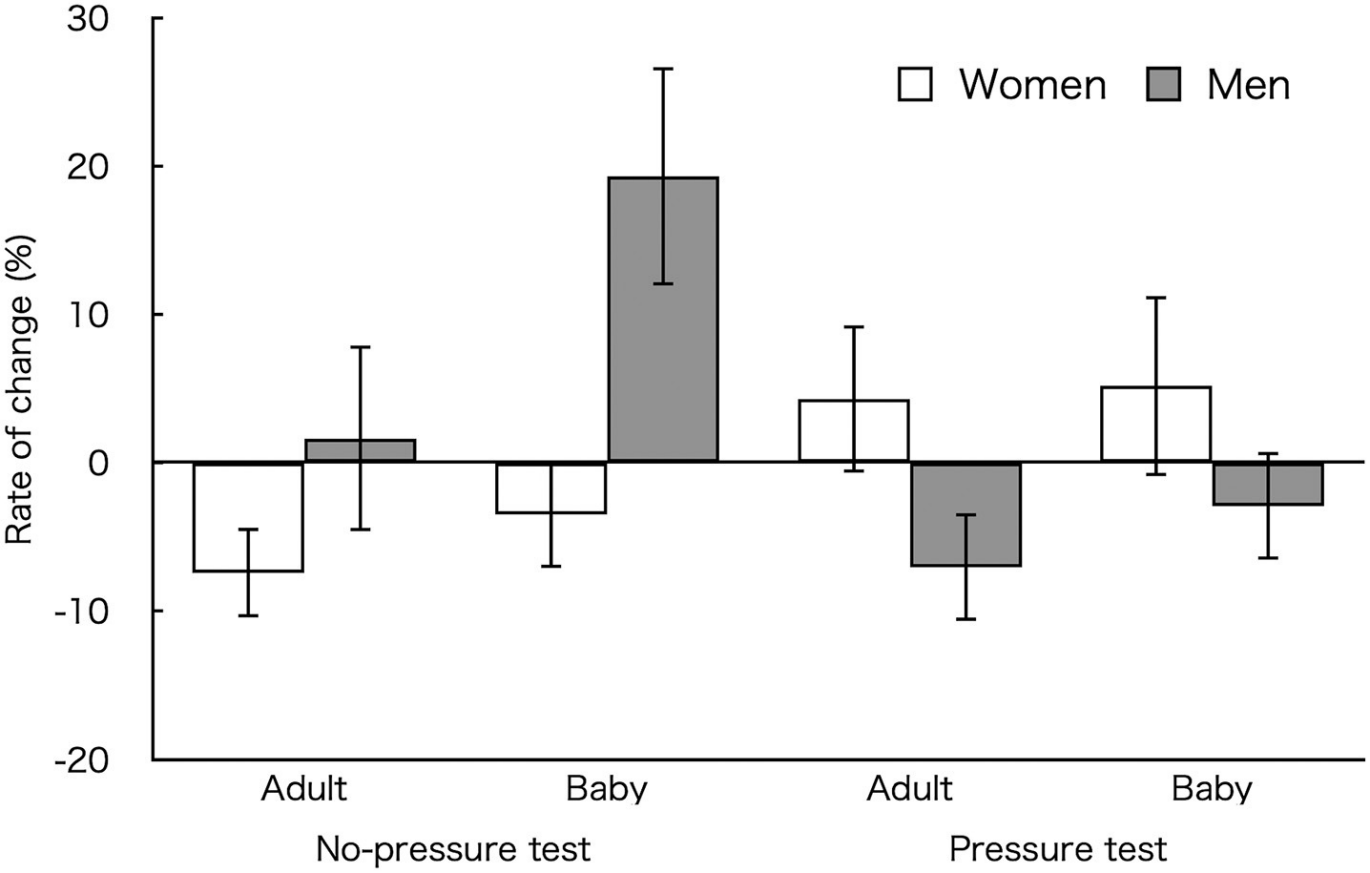
Change rate of percent accuracy in each picture-viewing condition relative to the control condition in each test. Error bars indicate standard error of the mean (SEM).

We also calculated the change rate of percent success in the baby and adult animal conditions relative to the control condition (Figure 2). A three-way ANOVA revealed a higher change rate in the baby (*M* ± *SEM* = 4.53 ± 2.76%) than in the adult animal condition (*M* ± *SEM* = −2.12 ± 2.19%) [*F*_(1, 23)_ = 6.54, *p* = 0.018, η_*p*_^2^ = 0.22]. An interaction between the group and picture conditions was significant [*F*_(1, 23)_ = 8.06, *p* = 0.009, η_*p*_^2^ = 0.26]. *Post-hoc* tests showed that the change rate was higher for male (*M* ±*SEM* = 10.48 ± 4.62%) than female participants (*M* ± *SEM* = −5.44 ± 4.09%) in the no-pressure test (*p* = 0.017, *d* = 1.04, 95% CI for Cohen's *d* [−0.19, 1.87]). In addition, male participants showed a higher change rate in the no-pressure (*M* ±*SEM* = 10.48 ± 4.62%) than in the pressure test (*M* ± *SEM* = −4.97 ± 4.65%; *p* = 0.032, *d* = 0.85, 95% CI for Cohen's *d* [0.14, 1.53]). An interaction between picture condition and test (pressure/no-pressure) reached significance [*F*_(1, 23)_ = 3.41, *p* = 0.078, η_*p*_^2^ = 0.13]. Two-way interaction was not found [*F*_(1, 23)_ = 1.35, *p* = 0.258, η_*p*_^2^ = 0.06].

A two-way ANOVA (Test × Picture) revealed neither a significant main effect [Test: *F*_(1, 24)_ = 0.17, *p* = 0.686, η_*p*_^2^ = 0.01, Picture: *F*_(2, 48)_ = 2.41, *p* = 0.101, η_*p*_^2^ = 0.09] nor an interaction [*F*_(2, 48)_ = 0.91, *p* = 0.408, η_*p*_^2^ = 0.04].

### State and Trait Anxiety

Table 2 shows the mean state anxiety scores (T-score) before each test. State anxiety was higher for female (*M* ± *SEM* = 45.63 ± 1.69) than male participants (*M* ± *SEM* = 39.09 ± 1.91) [*F*_(1, 23)_ = 6.59, *p* = 0.017, η_*p*_^2^ = 0.223]. Neither main effect of test [*F*_(1, 23)_ = 2.78, *p* = 0.109, η_*p*_^2^ = 0.11], nor interaction [*F*_(1, 23)_ = 0.39, *p* = 0.539, η_*p*_^2^ = 0.23] was significant. Table 3 shows the mean trait anxiety scores (T-score). Trait anxiety tended to be higher for female than male participants [*t*_(23)_ = 1.75, *p* = 0.093, *d* = 0.71, 95% CI for Cohen's *d* (−0.12, 1.51)].

**Table 2:** Mean scores of state anxiety before each test.

	**No-pressure test**		**Pressure test**	
	**Measured value *(SEM)***	**T-score *(SEM)***	**Measured value *(SEM)***	**T-score *(SEM)***
Women	39.21 (2.07)	43.44 (2.02)	43.71 (2.04)	47.83 (1.99)
Men	34.82 (2.49)	38.08 (2.38)	36.91 (2.81)	40.09 (2.69)

*SEM, standard error of the mean*.

**Table 3:** Mean scores of trait anxiety.

	**T-score *(SEM)***
Women	48.34 (2.34)
Men	41.66 (3.11)

*SEM, standard error of the mean*.

## Discussion

Using pressure manipulation, we investigated whether viewing cute pictures could improve the performance of basketball players during free throws. Although previous studies have reported that viewing cute pictures benefits performance in a fine-motor task (Sherman et al., 2009; Nittono et al., 2012; Yoshikawa et al., 2020) as well as other cognitive tasks (Nittono et al., 2012), to the best of our knowledge, this is the first study testing such beneficial effects in more dynamic sports skills.

We did not find a main effect for pictures, but we did find a three-way interaction. Female participants exhibited no change in free throw performance across any of the picture conditions, although they rated baby animals as cuter than adult animals. The null results were likely due to statistical power being too low to detect the cuteness-viewing effect. In contrast, male participants exhibited the beneficial effect of viewing cute pictures on free throw accuracy in the no-pressure test with a high effect size (Cohen's *d* = 0.86). It should be noted that the widely ranging 95% confidence intervals of the effect size (from −0.15 to 1.55) were likely due to the small sample size. Although the cuteness-viewing effect observed in male participants is an important and novel finding, further research is needed to confirm the existence of this beneficial effect by testing a larger number of participants.

The beneficial effect observed in male participants might be due to infantility that also underpins the cuteness-viewing effect (Nittono et al., 2012), because they rated infantility of baby animals higher than female participants. In contrast, our results could also be confounded by pleasantness of baby animals, which male participants also rated higher than female participants. Thus, induction of pleasantness might have partly contributed to our results, although pleasantness is not completely independent of cuteness.

It is fruitful to interpret our results from both a cognitive and an affective-motivational point of view by comparing them to those obtained by previous studies. The performance improvement after viewing cute pictures among male participants can be attributed to increased approach motivation, which must have narrowed the breadth of attentional focus (Gable and Harmon-Jones, 2008; Noguchi and Tomoike, 2016), and thus induced physical tenderness (Sherman et al., 2009) and caretaking behaviors (Griskevicius et al., 2010). One may raise the question of why female participants did not display the cuteness-viewing effect although they subjectively perceived stronger approach motivation for baby animal pictures than male participants. A previous study found that participants increased caretaking motivation as a function of the cuteness of baby face stimuli regardless of gender of viewers, but the trend was more prominent among female participants (Glocker et al., 2009). Thus, it is conceivable that the female individuals in our study increased their caretaking motivation by viewing cuteness. One possible explanation for the null result of viewing cuteness among female participants may be a ceiling effect. Based on their superior outcomes (the mean percent success across six conditions was 84%), it is reasonable to assume that the female participants had fully mastered shooting free throws; thus, it was difficult for them to improve their accuracy regardless of whether they viewed cute pictures. Our results may suggest that the cuteness-viewing effect might hardly occur for higher skilled athletes. Instead, the cuteness-viewing effect observed in male participants could be attributed to lower levels of expertise. In future studies, the inherent skill level of participants should be carefully controlled.

One may propose that the null result obtained in the case of female participants might be due to insufficient induction of pressure. There was no difference in state anxiety between the no-pressure and the pressure tests, which suggests that our pressure manipulation might not have adequately functioned. However, because male participants exhibited the cuteness-viewing effect only in the no-pressure condition, it is unlikely that the insufficient induction of pressure was responsible for the null result in female participants. If the pressure induction is a necessary condition for the cuteness-viewing effect, male participants' accuracy should not have improved in the no-pressure test. Our results suggest that the pressure induction might inhibit the cuteness-viewing effect, especially for less anxious individuals. Therefore, it is unlikely that the null result observed in female participants, who were more anxious than male participants, was due to insufficient pressure.

The result that no beneficial effect of viewing cute pictures was found in the pressure test is inconsistent with a previous study (Yoshikawa et al., 2020), in which the cuteness-viewing effect was detected even in a pressure situation. Interestingly, for male participants, the percent success in the control condition was greater during the pressure than the no-pressure test, suggesting an occurrence of clutch, defined as the phenomenon of performance improvement under pressure (Otten, 2009). However, for female participants, the percent accuracy in the control condition decreased more during the pressure than the no-pressure test, suggesting an occurrence of “choking under pressure” (Baumeister, 1984). Female participants exhibited higher state and trait anxiety than male participants in our experiment. Thus, these results can be interpreted to reflect an interactive effect among trait anxiety, state anxiety, and competitive ability.

One possible reason why the male participants did not exhibit the cuteness-viewing effect under pressure is the occurrence of clutch in the control condition that obscured the benefit of viewing cuteness. However, for the null result of female individuals in the pressure test, it is likely that deterioration was limited to the no-picture condition due to their expertise in shooting free throws. Thus, male participants who were mostly less anxious may have needed mild pressure to motivate themselves, whereas female participants who were mostly anxious decreased their performance although state anxiety was not increased by pressure manipulation. The current findings suggest that the beneficial effect of viewing cute pictures may be limited to a less pressure situation. However, our results cannot completely rule out the possibility that viewing cuteness can prevent choking under pressure. Further research is needed to clarify this issue.

Our results showed that viewing cute pictures may increase free-throw accuracy at least in a situation where inductive pressure is low and there is sufficient room for participants to improve. The cuteness-viewing effect in our study is reasonable from the perspective of an external focus of attention on sports skills (e.g., Wulf et al., 2010). Previous studies have confirmed that directing attention to external objects can facilitate automaticity in motor control and result in effective and efficient performance (Wulf et al., 2010). In a basketball free-throw task, accuracy was greater when participants attended the hoop (i.e., external focus) than when they attended wrist motion (i.e., internal focus) (Zachry et al., 2005). Because viewing cute pictures may facilitate the narrowing of attentional focus during the execution of a subsequent task (e.g., Gable and Harmon-Jones, 2008), a similar mechanism underlying the beneficial effects of external focus may be involved in the cuteness-viewing effect. Viewing cute pictures is likely one of the easiest techniques to induce external focus of attention as it does not require any technical intervention. Hence, it is necessary to adequately investigate the relationship between motor control and viewing cuteness in future research.

Recently, Yoshikawa et al. (2020) found that the gaze duration on the target, which is referred to as Quiet Eye (QE) duration (Vickers, 1996), was spontaneously prolonged after viewing baby animals in a no-pressure test. It would also be fruitful to consider the relationship between QE duration and the cuteness-viewing effect, although QE was not measured in our study. It is well known that skilled athletes have longer QE durations than novice athletes (Vickers, 1996). Additionally, athletes who choked under pressure exhibit a shorter QE duration than usual (Behan and Wilson, 2008; Wilson et al., 2009). Since longer QE is thought to represent proper attention control (Vine and Wilson, 2010, 2011; Vine et al., 2011) and sufficient motor programming (Vickers, 1996; Williams et al., 2002), athletes should prolong their QE durations before a critical movement to achieve good performance. According to the findings of Yoshikawa et al. (2020), simply viewing cute pictures that induce focal attention may prolong QE without any instruction-based intervention (i.e., QE training) in a low-pressure situation.

Our results suggest that viewing cute pictures may benefit athletes who should properly induce approach motivation before shooting free throws. Because the male participants did not show any changes in their subjective rating of excitement after viewing cute pictures, their improved performance cannot be explained by arousal. Some studies have reported that athletes benefit from viewing a personal motivation video in terms of enhancing motivation, confidence, and concentration (Halliwell, 1990; Tracey, 2011). These studies rely on the premise that the imagery and emotions induced by viewing a personal motivation video increase arousal to the optimum level in an inverted-U shape function (Yerkes and Dodson, 1908) before a sporting game (Dowrick, 1991, 1999). Contrary to such findings, viewing cute pictures appears to facilitate attention control without affecting arousal. Therefore, viewing cute pictures could lead to success in sporting skills that have a strong aversion to arousal, such as basketball free throws. Regarding practical implications, our findings may provide athletes with a new application that encourages them to view cute pictures printed on wrist bands, sportswear, tools, and so on immediately before the use of a critical skill and during a timeout, because viewing pictures requires neither a special skill nor a tool like the eye tracking system.

This study has some other limitations. First, the number of participants in our study was small. We could only test the small sample from the basketball teams that agreed with their participation in our experiment. Perhaps we should have adopted non-experienced players in terms of increasing power. On the other hand, it was still fruitful to investigate expert athletes, because the main purpose of this study was to investigate whether the cuteness-viewing effect can be obtained in a real sport skill. Further study testing numerous athletes would draw a clear-cut conclusion. Second, we did not use neutral pictures in the control condition in both the no-pressure and pressure tests. In comparing the animal and neutral picture viewing conditions, previous studies found performance improvements only after viewing baby animals (Sherman et al., 2009; Nittono et al., 2012) and never on adult animals and neutral pictures. Therefore, better free-throw accuracy would be more unlikely after viewing neutral pictures than adult animals, even if we used neutral pictures in the control condition. Third, because we recruited University athletes who were very busy with daily practice (i.e., 3-h trainings 6 days per week), we could not completely control for the experimental schedule; in fact, four of them participated in both the no-pressure and pressure tests on the same day. Nevertheless, it is still reasonable to accept the beneficial effects of viewing cuteness in the no-pressure test for male participants, which was fairly consistent with previous findings. It would also be intriguing to evaluate the differences in the cuteness-viewing effect by comparing players during the regular season and off-season. Since verification of the cuteness-viewing effect on sports performance has just begun, further research is expected to ameliorate the experimental protocol.

## Data Availability Statement

The raw data supporting the conclusions of this article will be made available by the authors, without undue reservation.

## Ethics Statement

The studies involving human participants were reviewed and approved by The Waseda University Ethics Committee. The patients/participants provided their written informed consent to participate in this study.

## Author Contributions

NY and HM contributed to the conception and design of the study. NY collected and analyzed the data. HM and NY drafted the manuscript. All authors contributed to the article and approved the submitted version.

## Conflict of Interest

The authors declare that the research was conducted in the absence of any commercial or financial relationships that could be construed as a potential conflict of interest.

## Footnotes

^1^For the power analysis (Faul et al., 2007), we input the following parameters: *f* = 0.25 (partial eta squared, medium = 0.06), α err prob = 0.05, 1-β err prob = 0.8, Number of groups = 2, Number of measurements = 6 (3 picture conditions × 2 pressure conditions), *Correlation among repeated measures* = 0.50, *Non-sphericity correction* ε = 1.
